# Structural barriers to antifungal drug development

**DOI:** 10.3389/fcimb.2026.1797104

**Published:** 2026-02-26

**Authors:** László Galgóczy

**Affiliations:** Department of Biotechnology and Microbiology, Faculty of Science and Informatics, University of Szeged, Szeged, Hungary

**Keywords:** antifungal agents, antifungal drug development, drug resistance, health equity, mycoses, research support

## Abstract

Fungal diseases represent a growing yet under-recognized global health threat, with mortality comparable to major infectious diseases but a disproportionately weak therapeutic pipeline. This review examines the antifungal innovation gap as a systemic phenomenon shaped by intertwined scientific, economic, and societal constraints. While multidrug-resistant pathogens underscore the urgent need for new antifungal agents, progress is hindered by fungal–human cellular similarity, high development costs, limited commercial incentives, and toxicity concerns. Agricultural fungicide practices that drive resistance and intellectual-property regimes that restrict affordability and generic entry further complicate this landscape. These scientific and economic barriers coexist with profound inequities in global access to existing antifungals, revealing a persistent gap between therapeutic availability and public health needs. The resulting public-health consequences include delayed treatment, reliance on suboptimal therapies, and widening disparities in outcomes across low-resource settings. This review proposes that antifungal agents should be conceptualized as global public goods and that non-profit development models offer a promising pathway to overcome current bottlenecks. An integrated perspective across clinical, agricultural, regulatory, and public-health domains underscores the need for coordinated strategies to restore innovation, ensure equitable access, and strengthen global preparedness against fungal diseases.

## 1 Introduction

Fungal infections cause substantial global morbidity and mortality. Recent World Health Organization (WHO) analyses indicate that approximately 3.8 million deaths are associated with invasive fungal infections annually, with around 2.5 million deaths directly attributable to fungal diseases, underscoring the magnitude and persistent under-recognition of this global health burden (World Health Organization, 2025). Estimates based on different methodological assumptions suggest that the global burden of fungal infections may be comparable to that of other major infectious diseases, such as malaria and tuberculosis. If accurate, this burden would position fungal infections among the most consequential yet under-recognized infectious threats worldwide (Denning, 2024). Many infections occur in patients with impaired immunity, including those receiving cancer chemotherapy, organ or stem cell transplantation, immunosuppressive therapy, or intensive care. Persons living with HIV and individuals with chronic lung disease are also at risk (Fisher et al., 2020; Denning, 2024; Anandani et al., 2025; Douvali et al., 2025; Van Rhijn and White, 2025). As global temperatures rise, environmental fungi are adapting to higher thermal tolerance, increasing the likelihood that new species will acquire traits enabling human infection (Rodrigues and Nosanchuk, 2020; Williams et al., 2024). Recent studies have demonstrated that exposure to mammalian body temperature can induce mutagenesis and promote the emergence of pan-drug-resistant and hypervirulent strains in environmental yeasts such as *Rhodosporidiobolus*, illustrating how thermal adaptation may facilitate the transition from environmental organisms to human pathogens (Huang et al., 2024). At the same time, multidrug-resistant pathogens such as *Candidozyma* (formerly *Candida*) auris and *Trichophyton indotineae* continue to spread in healthcare settings worldwide (Uhrlaß et al., 2022; Abdolrasouli et al., 2024; Choudhury et al., 2025; Zhang et al., 2025). These trends highlight the need for expanded antifungal treatment options.

However, antifungal drug development is slow. Only a few new classes of antifungal agents have been introduced in the past four decades (Puumala et al., 2024; Galgóczy, 2025). This structural stagnation is reflected in the global antifungal pipeline. According to the WHO, only four new antifungal drugs have been approved by major regulatory agencies in the past decade, and the current clinical pipeline remains sparse and insufficient to address priority fungal pathogens (World Health Organization, 2025). Many compounds under investigation fail during early development because fungal pathogens are eukaryotic organisms, making selective targeting difficult and increasing the risk of toxicity (Scorzoni et al., 2017; Puumala et al., 2024). Similar to antibiotics, antifungal treatments also have limited commercial potential because they are used for short durations and in relatively small patient populations compared with therapeutic areas such as oncology or cardiovascular disease (Myung and Klittich, 2015; Scorzoni et al., 2017; Årdal et al., 2020). These characteristics contribute to a persistent gap between public health needs and available antifungal therapies.

This review examines the societal and ethical factors underlying this gap and discusses why new approaches (including non-profit models) may be needed to address a growing global health threat.

## 2 Structural drivers of the antifungal innovation gap

### 2.1 Neglected global threats

Fungal diseases disproportionately affect populations with fewer healthcare resources. These include persons living in low- and middle-income countries, individuals without access to timely diagnosis, and patients with immunocompromising conditions (Mudenda, 2024). Many at-risk communities have limited economic leverage in global pharmaceutical markets. As a result, diseases with high mortality but limited commercial potential receive less attention in research and development (R&D). Despite a mortality burden similar to major infectious diseases, fungal pathogens remain underrepresented in global research investments (Rodrigues and Nosanchuk, 2020; Smith et al., 2023; Lappé et al., 2024). Compared with bacterial or viral infections, fungal infections receive a smaller share of public funding, philanthropic support, and commercial interest. This imbalance contributes to slower progress in developing diagnostic tools, surveillance systems, and therapeutic options. Under-recognition of fungal diseases in global health strategies also affects preparedness and response efforts.

The combination of high disease burden, limited investment, and uneven access to diagnostics and care creates substantial challenges for national health systems. Addressing fungal diseases requires attention to these structural disparities and recognition that fungal infections represent an emerging threat in global health.

### 2.2 Technological and economic barriers to antifungal innovation

Antifungal research presents unique scientific and economic challenges. Fungi share many cellular pathways with humans, so identifying targets that are effective against fungi but safe for human cells is complex (Scorzoni et al., 2017; Puumala et al., 2024). These scientific barriers increase the cost and uncertainty of drug development. In addition, antifungal agents are typically administered for short durations, reducing expected sales revenue relative to therapies for chronic conditions. The small market size, combined with high research expenditures, discourages private-sector investment (Niño-Vega et al., 2024; Puumala et al., 2024; Wolfgruber et al., 2024).

In addition to economic constraints, antifungal R&D faces practical technological bottlenecks at the earliest stages of discovery. Target identification and validation remain difficult because many essential fungal pathways are conserved in humans, narrowing the space for selectively druggable targets with acceptable safety margins (Scorzoni et al., 2017; Puumala et al., 2024). Even when promising targets are proposed, robust genetic validation, pathway redundancy, and species-to-species differences in target essentiality can limit generalizability across priority pathogens, complicating efforts to develop broadly useful agents (Puumala et al., 2024; Li et al., 2025).

Screening capacity also remains a constraint. Compared with antibacterial discovery, antifungal screening is often limited by species-specific growth requirements, slower growth kinetics, and the need to capture clinically relevant phenotypes such as filamentation, stress adaptation, and biofilm-associated tolerance. As a result, many campaigns rely on relatively simple growth inhibition readouts, which may miss antivirulence or morphology-specific vulnerabilities and can yield hits with limited breadth across fungal taxa (Calderone et al., 2014; Puumala et al., 2024). Recent efforts to develop broader, standardized phenotypic screening approaches highlight both progress and the continued need for scalable, reproducible assays that enable cross-species comparability early in discovery (Calderone et al., 2014; Puumala et al., 2024).

Downstream translation introduces additional attrition. Antifungal candidates frequently fail due to narrow therapeutic windows, suboptimal tissue penetration at sites of invasive disease, drug-drug interactions in complex patient populations, and uncertainty in predicting *in vivo* efficacy from *in vitro* activity. These challenges are amplified by the limited availability of clinically informative preclinical models and the operational difficulty of conducting adequately powered trials for relatively uncommon, but high-mortality infectious syndromes (Puumala et al., 2024; World Health Organization, 2025). Consistent with this, WHO pipeline assessments indicate that the current candidate pool remains insufficient in number and diversity relative to global priorities, and that truly innovative candidates are still limited (World Health Organization, 2025).

To partially circumvent pathogen-intrinsic resistance mechanisms and narrow target space, interest has grown in adjunctive approaches that do not rely exclusively on direct fungal killing. Host-directed and immunomodulatory strategies have been discussed as potential complements to conventional antifungal therapy, particularly for invasive disease in immunocompromised hosts (Giannella et al., 2025). In parallel, targeted delivery and formulation innovation aim to improve exposure at infection sites while reducing systemic toxicity, an especially relevant challenge for polyenes such as amphotericin B. Recent work has summarized advances in nanotechnology-enabled and carrier-based delivery systems designed to enhance antifungal activity and tolerability, underscoring both promise and translational barriers such as manufacturability, regulatory complexity, and safety assessment (Zadeh Mehrizi et al., 2024). Together, these technological limitations interact with economic disincentives to constrain the pace of antifungal innovation.

Together, these factors contribute to persistent structural barriers across scientific, economic, and public-health domains (**Table 1**). WHO pipeline analyses corroborate this assessment, showing that the number, diversity, and innovation potential of current antifungal candidates remain inadequate relative to the burden posed by priority fungal pathogens (World Health Organization, 2025). In response to these constraints, growing interest has emerged in alternative innovation strategies, including drug repurposing and optimization of existing compounds, which aim to circumvent the high costs and risks of *de novo* antifungal drug development (Donlin and Meyers, 2022).

**Table 1:** **│** Major barriers to antifungal innovation by category.

Category	Key drivers	Public-health impact
Market failure	Limited commercial viability High research and development cost	Few novel antifungal drugs developed
Resistance development	Emergence of multidrug-resistant strains Azole fungicide use in agriculture	Reduced treatment options
Inequitable access	High cost of newer antifungals Limited availability in low-resource regions	Disparities in treatment availability
Agriculture	Azole fungicide selection pressure Environmental spread of resistant fungi	Compromised effectiveness medical azoles
Intellectual property	Patent protection Delayed entry of generics	Limited affordability of antifungal therapies

Because antifungal agents are essential for treating life-threatening infections, limited commercial interest creates a public health gap. When market incentives are insufficient to support needed development, governments and public health organizations may need to intervene. The trend toward reduced commercial involvement in antifungal research suggests that the traditional pharmaceutical model may be inadequate for meeting current and future needs. Characterizing antifungal agents as global public goods can help guide policy strategies that address this gap.

### 2.3 Antifungal resistance

Antifungal resistance is a growing concern worldwide. Resistance can develop during clinical treatment when pathogens are repeatedly exposed to antifungal drugs (Fisher et al., 2022; Verma et al., 2023; Hui et al., 2024; Niu et al., 2024). It can also arise in the environment. A notable example is the emergence of azole-resistant *Aspergillus fumigatus* due to agricultural use of azole fungicides, which are structurally similar to medical triazoles (Verweij et al., 2009; Snelders et al., 2012). Resistant strains can cause infections that no longer respond to first-line azole therapy. Resistance complicates treatment decisions and clinical outcomes, often necessitating alternative therapies that may be less effective, more toxic, or unavailable in many settings. Surveillance studies show an expanding geographic range of resistant fungal pathogens, including *C. auris*, which has caused outbreaks in healthcare facilities on multiple continents (Choudhury et al., 2025; Zhang et al., 2025). Dermatophytes such as *T. indotineae* have also developed widespread terbinafine resistance, creating new challenges for treatment (Hui et al., 2024). In addition to acquired resistance, several clinically important fungi exhibit intrinsic or multidrug resistance. For example, *Lomentospora prolificans* is characterized by broad resistance to most currently available antifungal classes, which severely limits therapeutic options and is associated with poor clinical outcomes (Konsoula et al., 2022). Furthermore, resistance may emerge through adaptive physiological responses that precede stable genetic changes. Experimental and clinical studies have described stress-induced or priming responses, such as triazole priming in *A. fumigatus*, in which transient antifungal exposure promotes physiological states that facilitate the later emergence of stable resistance phenotypes (Harish et al., 2022). This phenomenon illustrates that antifungal resistance may arise through stepwise adaptive processes and not exclusively through classical mutation-driven mechanisms (Fisher et al., 2022; Verma et al., 2023). These adaptive mechanisms add further complexity to antifungal resistance evolution and treatment outcomes.

Addressing antifungal resistance therefore requires coordinated action across clinical, agricultural, and environmental sectors, including strengthened surveillance, improved stewardship programs, and enhanced regulatory approaches to agricultural fungicide use. Resistance trends also underscore the urgent need for new antifungal drug classes and improved access to existing treatments. In this context, WHO evaluations indicate that only a minority of current clinical candidates meet innovation criteria such as novel targets, new chemical classes, or absence of cross-resistance, highlighting the limited capacity of the existing pipeline to counter multidrug-resistant fungi (World Health Organization, 2025).

### 2.4 Inequitable access

Access to antifungal therapy varies widely across regions. While some newer antifungals, such as ibrexafungerp and rezafungin, are costly and may be unavailable in low-resource settings (Rodrigues and Nosanchuk, 2020; Robertson et al., 2023; Branda et al., 2025), global analyses have shown that disparities in availability and cost also affect long-established essential antifungal drugs across countries and healthcare systems (Kneale et al., 2016). Limited availability affects patient outcomes, especially in areas with high burdens of HIV-associated fungal infections and respiratory fungal diseases. When treatment options are restricted by cost, patients may experience delays in care, use older and more toxic medications, or receive no treatment at all. These disparities highlight concerns about equitable healthcare access. Regions with the highest fungal burdens often lack affordable antifungal options, diagnostic capacity, and essential infrastructure. These challenges can increase mortality and contribute to continued transmission of resistant strains.

Improved access depends on several factors, including global pricing models, procurement strategies, regulatory harmonization, and expansion of non-profit development pathways that emphasize affordability.

### 2.5 Agricultural–medical conflicts

Agricultural practices influence the effectiveness of medical antifungals. Triazole fungicides are widely used in crop protection because they prevent major agricultural losses. However, their extensive use creates selective pressure in the environment for azole-resistant *A. fumigatus*, a phenomenon now widely recognized as a One Health issue linking agricultural fungicide use to clinical resistance (Verweij et al., 2009; Snelders et al., 2012; Castro-Ríos et al., 2025; EFSA, 2025). These resistant strains can spread to humans and lead to infections that do not respond to standard therapy.

This cross-sector interaction raises important questions about how agricultural and public health priorities are balanced. Although some regions have implemented guidelines to monitor fungicide use, regulatory frameworks vary, and enforcement is inconsistent. More coordinated approaches are needed to ensure that agricultural practices do not compromise human health outcomes. Public health partnerships with agricultural agencies can support risk assessments, promote safer fungicide alternatives, and develop strategies to limit the emergence of resistant environmental strains.

### 2.6 Intellectual-property barriers

Intellectual-property (IP) protections shape access to antifungal drugs. Patent exclusivity can lead to high prices and limited availability, especially in low-resource settings (Tenni et al., 2022). Delayed entry of generic formulations also affects affordability (Yao, 2024). Because research priorities often follow commercial incentives, diseases with smaller markets may receive less attention, contributing to limited innovation (Chapman et al., 2024; Mwoka, 2024). Revisiting IP strategies could help support the development and distribution of antifungal agents. Approaches such as patent pooling, non-exclusive licensing, or open-science frameworks may promote broader access while maintaining incentives for innovation (Burrone et al., 2019; Medicines Patent Pool, 2023; European Parliament, 2023). These strategies have been successfully implemented in other disease areas and could be adapted for antifungal development.

## 3 System-level responses to the antifungal innovation gap

### 3.1 Non-profit development models

Alternative approaches to drug development are increasingly important for antifungal research. Non-profit organizations such as the Drugs for Neglected Diseases Initiative have shown that essential medicines can be developed through models that prioritize health needs rather than market profitability (Drugs for Neglected Diseases initiative, 2024). These organizations emphasize affordability, equitable access, and collaboration across sectors.

A coordinated global antifungal pipeline could adopt similar principles. WHO analyses highlight persistent financial and technical limitations in current antifungal R&D and call for coordinated strategies to address unmet medical needs, thereby providing institutional justification for alternative, public-interest-driven development models (World Health Organization, 2025). Building on this rationale, a global antifungal pipeline could integrate early research, preclinical testing, clinical trial networks, and manufacturing support into a unified framework involving governments, academic institutions, international agencies, and non-profit partners. Conceptualizing antifungals as global public goods offers a normative foundation for such efforts, emphasizing shared benefits, sustainable access, and collective responsibility for preserving the effectiveness of essential medicines.

### 3.2 Role of governments and public institutions

Governments and international health organizations play critical roles in antifungal preparedness. Their responsibilities include surveillance of emerging infections, investment in research, stewardship programs, and regulatory oversight of agricultural fungicide use (Hui et al., 2024; World Health Organization, 2025). Public-sector support can help address market gaps and ensure that high-priority pathogens receive adequate research attention. Investing in R&D is essential for addressing emerging threats. Funding from national agencies, multilateral organizations, and public–private partnerships supports innovation and capacity building. Governments also help define regulatory pathways and ensure that essential medicines remain available and affordable (Bertagnolio et al., 2024; World Health Organization, 2025; Lu et al., 2025).

Without coordinated public-sector involvement, global capacity to prevent and treat fungal infections is limited. Ensuring that antifungal strategies are integrated into national and international health plans is an important step toward strengthening global health security.

### 3.3 Global initiatives mitigating the antifungal innovation gap

International organizations have begun to address the growing disconnect between fungal disease burden and the limited pipeline of antifungal therapeutics. Two major efforts, the Global Action for Fungal Infections (GAFFI) and the WHO’s fungal priority pathogens list (FPPL), provide frameworks for improving diagnosis, treatment access, surveillance, and research investment on a global scale.

GAFFI, established in 2013, focuses on reducing deaths and disability caused by fungal diseases through improved access to diagnostics, essential medicines, and public-health policy development (Denning, 2015). Its initiatives emphasize rapid diagnostic scale-up, particularly in low-resource settings where mortality from diseases such as cryptococcal meningitis, *Pneumocystis* pneumonia, and disseminated histoplasmosis remains high (Denning, 2015; Bongomin et al., 2017). GAFFI advocates for wider availability of essential antifungal medicines, including amphotericin B, flucytosine, and itraconazole, whose access remains inconsistent in many low- and middle-income countries despite their inclusion on the WHO Essential Medicines List (Bongomin et al., 2017). By identifying gaps in access and supporting regional training programs, GAFFI strengthens national capacities and provides evidence that can guide governmental and donor-driven investment.

The WHO FPPL, first released in October 2022, represents the earliest formal prioritization of fungal pathogens by global health importance. The list categorizes 19 fungal pathogens into “critical,” “high,” and “medium” priority tiers based on factors such as mortality, incidence, resistance, diagnostic difficulty, and unmet research needs (World Health Organization, 2022). *Candida albicans*, *C. auris*, azole-resistant *A. fumigatus*, and *Cryptococcus neoformans* were placed in the highest-priority tier, highlighting the urgent need for new diagnostics and therapeutics. The FPPL explicitly identifies deficiencies in the antifungal pipeline, calling for increased public-sector investment, improved surveillance systems, and strengthened regulatory pathways to accelerate innovation (World Health Organization, 2022).

Together, GAFFI and the WHO FPPL provide complementary mechanisms to address the antifungal innovation gap: GAFFI drives implementation and access at clinical and national levels (Denning, 2015), while the FPPL sets global research and policy priorities. WHO pipeline analyses further demonstrate that current antifungal development efforts remain insufficient to meet global health priorities, underscoring the need for coordinated international action (World Health Organization, 2025). Collectively, these frameworks help reorient antifungal development toward a public-health paradigm and strengthen the rationale for non-profit or public-interest innovation models. By articulating unmet needs and mobilizing stakeholders, they play a critical role in mitigating the consequences of limited commercial investment and enhancing resilience against emerging fungal threats.

### 3.4 Transparency and trust

Transparency in research, reporting, and decision-making is central to effective public health action. Studies have shown that industry sponsorship can influence research outcomes (Lundh et al., 2017; Siena et al., 2023). Selective reporting or delayed publication of data related to resistance or drug safety can hinder timely policy responses (Grobusch et al., 2024; Morrow et al., 2022). Transparent evidence sharing supports public trust, informs regulatory decisions, and contributes to appropriate clinical and agricultural stewardship. Increasing open access to research findings, improving reporting standards, and encouraging independent evaluations can strengthen antifungal research and support effective global responses.

## 4 Conclusion

**Figure 1:**
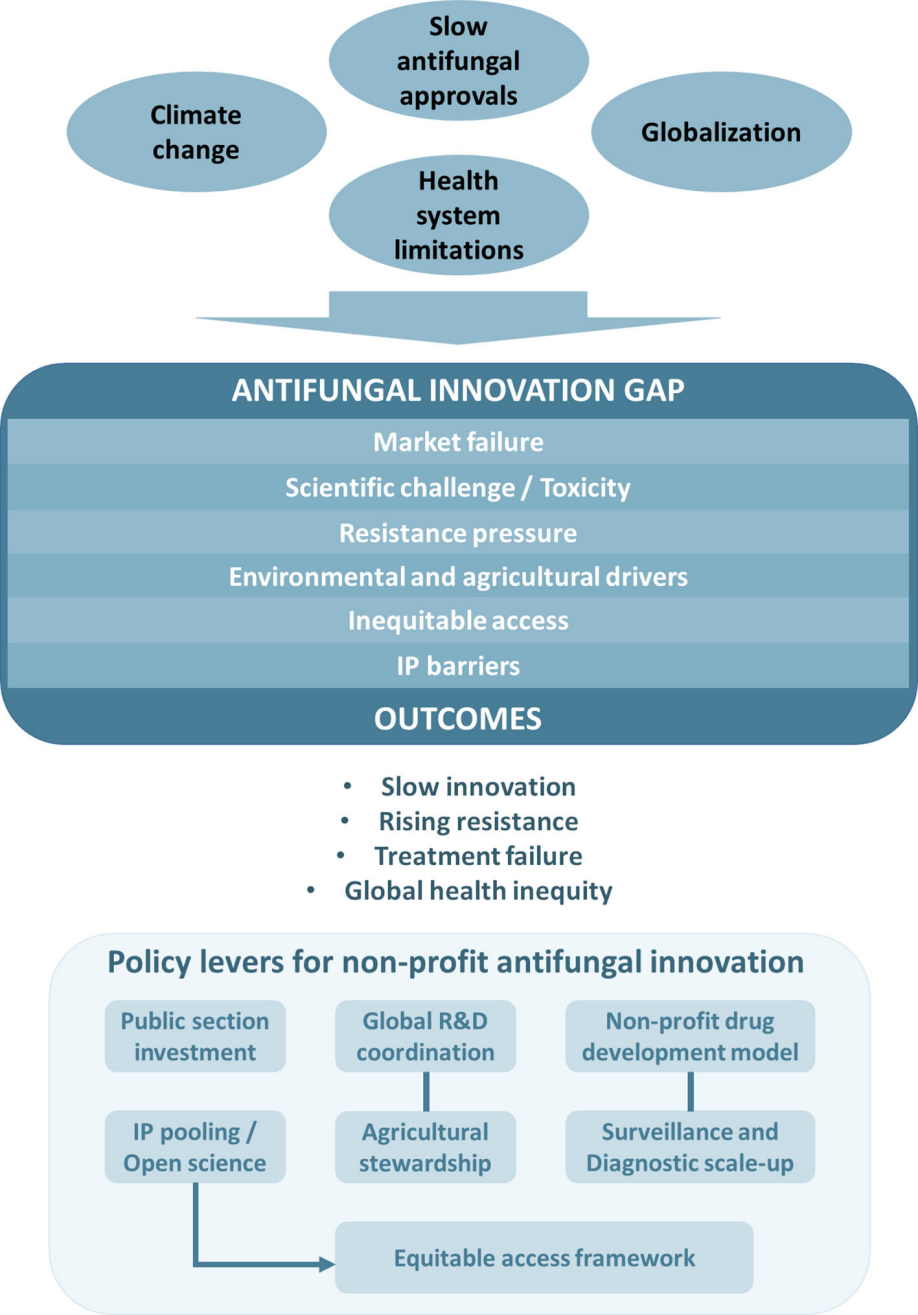
Conceptual framework illustrating the drivers and consequences of the antifungal innovation gap and key policy levers for non-profit antifungal development. Multiple global forces (including climate change, globalization, health-system limitations, and slow antifungal discovery) contribute to an expanding antifungal innovation gap. Core components of this gap include market failure, scientific and toxicity barriers, resistance pressures, environmental and agricultural drivers, inequitable access, and intellectual-property constraints. These factors collectively result in slow innovation, rising antifungal resistance, treatment failure, and widening global health inequities. The lower panel outlines potential policy levers to address these challenges, emphasizing the roles of public-sector investment, global R&D coordination, non-profit drug-development models, IP pooling and open-science mechanisms, agricultural stewardship, and strengthened surveillance and diagnostics. Together, these actions support equitable access frameworks and a more resilient antifungal R&D ecosystem.

Antifungal drug development is constrained by scientific complexity, limited commercial incentives, emerging resistance, and persistent inequities in access. These interrelated barriers compromise patient outcomes and global health preparedness. Addressing them requires integrated action across clinical, agricultural, environmental, and regulatory domains. Non-profit development models, expanded public-sector investment, and strengthened global surveillance offer viable pathways to overcome gaps left by market-driven pharmaceutical systems. Conceptualizing antifungal therapies as essential global public goods provides a normative foundation for strategies that enhance access, preserve drug effectiveness, and strengthen global capacity to respond to fungal threats. In line with the conceptual framework presented here (**Figure 1**), such a shift demands systemic reforms in innovation, governance, and access. WHO assessments of the antifungal pipeline confirm that current research and development efforts remain insufficient to meet the needs posed by priority fungal pathogens, underscoring the urgency of coordinated global action (World Health Organization, 2025).
